# Nephrotic Syndrome Without Nephrotic Range Proteinuria

**DOI:** 10.7759/cureus.64342

**Published:** 2024-07-11

**Authors:** Jibran A Sheikh, Usheem Syed, Sayed M Osama

**Affiliations:** 1 Internal Medicine, Central Michigan University College of Medicine, Saginaw, USA; 2 Nephrology, Central Michigan University College of Medicine, Saginaw, USA

**Keywords:** systemic amyloidosis, nephrotic-range proteinuria, albumin clearance, hepatic amyloidosis, nephrotic syndrome

## Abstract

Nephrotic syndrome in adults is defined as nephrotic-range (≥3.5g/24h) proteinuria with low serum albumin, usually associated with edema, hyperlipidemia, and lipiduria. The 3.5g/24h threshold was selected arbitrarily and might not be reached in certain cases despite severe defects in glomerular permeability. We describe the case of a 57-year-old male who presented with progressively worsening swelling involving his limbs and abdomen. He also reported decreased urine output and fatigue. Physical examination was notable for severe pitting edema over legs, arms, and abdomen, in addition to peri-orbital puffiness. Labs revealed low serum albumin (1.3 g/dL), moderate proteinuria (2.3g/24h), and elevated total cholesterol (334 mg/dL). Renal biopsy showed amyloid light chain (AL) amyloidosis and bone marrow biopsy confirmed the presence of lambda-restricted plasma cells. Computed tomography, ultrasound, elastography, and laboratory findings were congruent with those seen in hepatic amyloidosis. A diagnosis of nephrotic syndrome caused by systemic AL amyloidosis was made despite the absence of nephrotic range proteinuria. The primary abnormality in nephrotic syndrome is increased glomerular permeability, leading to severe proteinuria causing low serum albumin, decreased oncotic pressure, and increased water retention by kidneys due to activation of the epithelial sodium channel (ENaC). The amount of albuminuria is influenced by both the extent of glomerular permeability and the rates of glomerular filtration and albumin synthesis. In cases where albumin synthesis is decreased secondary to concurrent liver disease, as in our case, a steady state of renal protein excretion may be reached at a lower threshold than 3.5g/24h despite severe defects in glomerular permeability.

## Introduction

Nephrotic syndrome as a constellation of symptoms of profuse albuminuria, hypo-albuminemia, and edema resulting from diseased kidneys leaking protein into the urine was established by the early 19th century [1]. And by the middle of the 20th century, a consensus definition of nephrotic syndrome, then known as lipid nephrosis, also came into being [2]. It was described as “a syndrome of proteinuria, principally albuminuria; hypoproteinaemia; hypercholesterolemia; lipiduria; and edema in the form of anasarca and effusions”. At that time, there was no widely agreed-upon numerical criterion used to differentiate between proteinuria categorized as 'nephrotic' and 'non-nephrotic'. It was not until 1958 that a widely accepted quantitative definition of nephrotic syndrome was presented by Berman and Schreiner [3], as the identification of excreting 3.5 g or more total protein per day, accompanied by the presence of doubly refractile 'oval fat' bodies in urine sediment. However, no detailed justification was provided for this arbitrary threshold of proteinuria used to define nephrotic syndrome. Also, their study was “not prospectively designed to establish a ‘diagnostic’ threshold nor powered sufficiently to give it credence” [4].

Despite these potential limitations, the threshold of 3.5 g of protein excretion per day has remained central to the definition of nephrotic syndrome in adults to date. The simplicity of a single value and sensitivity of this threshold to capture most nephrotic syndrome cases has led to the persistence of this rather arbitrarily selected value in current clinical practice. But, like every other diagnostic test and criteria, the sensitivity of this threshold is far from perfect and should not be excessively relied upon [4, 5]. We present a case of nephrotic syndrome with proteinuria < 3.5 g/24h.

## Case presentation

A previously healthy 57-year-old male presented with progressively worsening swelling involving his limbs and abdomen ongoing for the past six months. He also reported decreased urine output, fatigue, and early satiety due to abdominal swelling. He denied dyspnea, abdominal pain, hematuria, rash, or fever.

At presentation, his oral temperature, heart rate, respiratory rate, blood pressure, and oxygen saturation on room air were 97.6°F, 116 beats/min, 20 breaths/min, 97/84 mmHg, and 98% respectively. Physical examination was notable for severe pitting edema over legs, arms, and abdomen, in addition to peri-orbital swelling. Lungs were clear to auscultation with decreased sounds at the lung bases; the abdomen was non-tender and distended with a positive fluid thrill. Jugular venous pressure was not elevated and there was no skin rash. Initial labs are given in Table 1.

**Table 1:** Serum and urine laboratory values at admission. Abbreviations: ALT, alanine transaminase; ALKP, alkaline phosphatase; AST, aspartate transaminase; BUN, blood urea nitrogen; eGFR, estimated glomerular filtration rate; GGT, gamma-glutamyltransferase; LDL, low-density lipoprotein; RBC, red blood cell; WBC, white blood cell.

	Value	Normal range
Serum Chemistry		
Sodium (mmol/L)	133	136-145
Potassium (mmol/L)	4.2	3.5-5.1
BUN (mg/dL)	19	8-25
Creatinine (mg/dL)	0.9	0.7-1.2
eGFR (mL/min)	105	
Albumin (g/dL)	1.3	3.2-4.6
Protein (g/dL)	4.9	6-8.3
AST (U/L)	80	5-34
ALT (U/L)	30	0-55
ALKP (U/L)	1193	40-150
GGT (U/L)	670	12-64
Bilirubin (mg/dL)	0.8	0.3-1.2
Total Cholesterol (mg/dL)	334	0-200
LDL (mg/dL)	284	< 130
Triglycerides (mg/dL)	101	< 150
Urine Chemistry		
RBC (/hpf)	<5	<5
WBC (/hpf)	<5	<5
24h urine protein (g/24h)	2.3	50-80
Protein/Creatinine Ratio (mg/mg)	2.3	< 0.1
Dipstick Protein	>300 mg/dL	Negative

**Figure 1:**
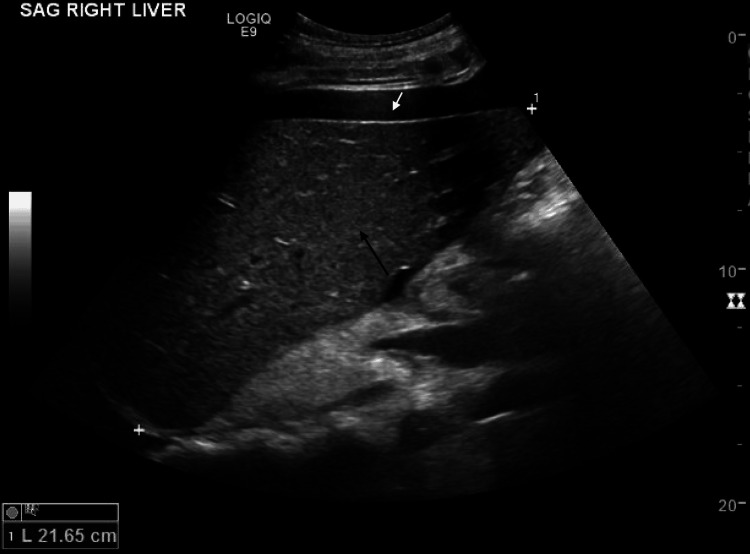
Ultrasound of the liver (sagittal) showing heterogeneous echogenicity (black arrow), ascites (white arrow), and hepatomegaly (cranio-caudal dimension 21.65 cm).

**Figure 2:**
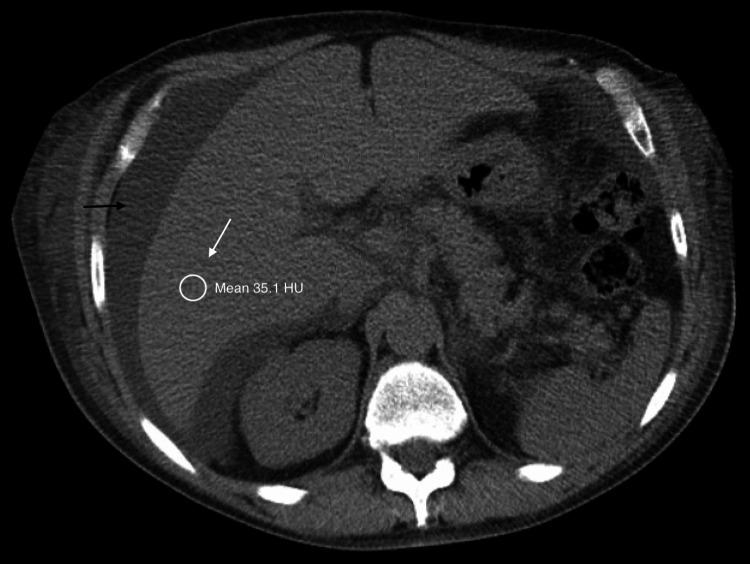
Non-contrast CT abdomen showing ascites (black arrow) and diffusely decreased hepatic attenuation (white arrow). Abbreviations:  HU, Hounsfield unit.

Transthoracic echocardiography was unremarkable. Liver and biliary imaging with ultrasound, non-contrast computed tomography (CT) and MRI were also unremarkable except for heterogeneous echogenicity on ultrasound and diffuse decreased parenchyma attenuation on CT in addition to hepatomegaly and mild ascites (Figures 1, 2). Two-dimensional shear wave elastography of the liver showed a V median of 8.81 kPa suggestive of mild to moderate liver fibrosis. 

**Figure 3:**
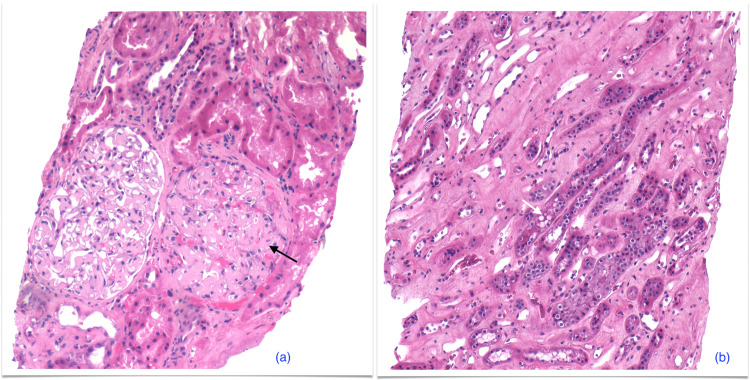
Renal biopsy, hematoxylin and eosin: (a) The glomeruli show expansion of the mesangium and thickened glomerular capillary loops with deposition amorphous material (black arrow). (b) Protein reabsorption droplets are seen within tubular epithelial cells (white arrow). There is focal deposition of amorphous material in the interstitium.

Renal biopsy with immunofluorescence showed lambda amyloid light chain (AL) amyloidosis, confirmed by mass spectrometry. Biopsy findings on hematoxylin and eosin (H&E) staining are shown in Figure 3. No monoclonal proteins were seen on serum electrophoresis but immunofixation showed faint bands of immunoglobulin G (IgG) kappa and lambda. Free kappa (80 mg/L, normal 3.3-19.4 mg/L) and lambda chain (270 mg/L, normal 5.7-26.3 mg/L) levels were elevated but the ratio was normal (0.3, normal 0.26-1.65). Bone marrow biopsy and immunostaining confirmed the presence of lambda-restricted plasma cells making up 0.4% of viable leucocytes.

A diagnosis of nephrotic syndrome caused by systemic AL amyloidosis secondary to plasma cell neoplasm was made. The patient was not found to be a candidate for stem cell transplantation and hence was managed with bortezomib-based chemotherapy.

## Discussion

The primary abnormality in nephrotic syndrome is increased glomerular permeability, leading to severe albuminuria causing low serum albumin, decreased oncotic pressure, and increased water retention by kidneys due to activation of epithelial sodium channel (ENaC) [6]. The degree of albuminuria is dependent not only on the degree of glomerular permeability but also on other factors such as glomerular filtration rate (GFR) and serum albumin concentration [4,5].

Rarely, patients with nephrotic syndrome experience severe hypoalbuminemia (e.g., <2.0 g/dL) while exhibiting protein excretion rates of less than 3.5 g per day. In cases of sustained but heightened glomerular permeability, proteinuria diminishes as albumin levels decline until a balance is achieved between synthesis and excretion. The extent of glomerular permeability impairment in these patients can be demonstrated by increasing plasma albumin levels through intravenous infusion of hyper-oncotic human serum albumin. With sufficient infusion, urine protein excretion rates can rapidly escalate to significant levels (e.g., >20 g/day), and the infused albumin is completely excreted within 24-48 hours. This problem might be addressed by employing urinary albumin clearance rather than a fixed total protein excretion threshold in defining nephrotic syndrome [4]. The urinary albumin clearance, in this case, should also be “adjusted for the GRF [urinary albumin excretion rate (in mg/24 h) divided by the serum albumin concentration (in mg/mL) corrected to an average GFR of ∼140 L per 24 h (∼100 mL/min) in an adult] with a value for urinary albumin clearance (GFR adjusted) of about >100 mL/24 h or greater representing a nephrotic-range of albumin clearance” [4].

This patient had all the other features of nephrotic syndrome (severe hypoalbuminemia, hypercholesterolemia, anasarca, and biopsy-proven renal amyloidosis) despite not having nephrotic range proteinuria. Though the GFR was normal, decreased hepatic synthesis likely contributed to low serum albumin levels, leading to the steady state of renal albumin excretion being achieved at a lower threshold than 3.5 g/day despite the severe increase in glomerular permeability. This could have been confirmed by estimating albumin clearance by the above-given equation or by quantifying albuminuria after albumin infusions. We could not calculate the albumin clearance precisely as the value of urinary albumin was not obtained during the hospitalization. Assuming the percentage of albumin in the urinary protein was at least > 60%, the estimated albumin clearance corrected for the normal GFR would be > 100 mL/min [(0.6 x 2.3 / 1.3) (100/105)] and hence would qualify for nephrotic range albumin clearance.

The reason for decreased albumin synthesis, in this case, can be attributed to the hepatic involvement from the AL amyloidosis. Isolated elevations in gamma-glutamyl transferase (GGT) and alkaline phosphatase (ALKP) with associated imaging findings of hepatomegaly, diffuse hypoattenuation on CT, diffuse increase in liver echogenicity on ultrasound and increased liver stiffness on elastography, as present in this patient, have been characteristically described in hepatic amyloidosis [7,8]. 

In healthy adults, the albumin synthesis rate of about 0.15 g/kg/day (10.5 g/day for 70 kg adult) is balanced by renal (≈6%), gastrointestinal (≈10%), and catabolic (≈84%) clearances. Albumin synthesis increases with increasing loss of albumin in the urine and as the urine albumin excretion rate increases to a maximal value of 0.24 g/kg/day (16.8 g/day/70 kg), total albumin synthesis approximately doubles, increasing by about 0.16 g/kg/day (11.2 g/day for a 70 kg adult) and the total urine albumin excretion increases to about 78% of the total synthesis rate. However, in liver disease, the rate of albumin synthesis decreases in proportion to the decrease in serum albumin concentrations [9]. When significant liver disease co-exists with nephrotic syndrome, the liver cannot increase its synthesis of albumin, which rather may be decreased. This limitation in the rate of albumin synthesis limits the amount of urinary albumin excretion that is possible even when the glomerular permeability is severely impaired.

## Conclusions

This case highlights a rare presentation of nephrotic syndrome secondary to systemic AL amyloidosis, characterized by severe hypoalbuminemia, generalized edema, and biopsy-confirmed renal amyloid deposits despite proteinuria being below the traditional nephrotic range threshold of 3.5 g/24 hours. The patient's clinical course underscored the limitations of relying solely on total protein excretion as a diagnostic criterion for nephrotic syndrome, especially in cases where significant hypoalbuminemia affects the urinary albumin excretion rate. This report emphasizes the importance of considering additional parameters such as albumin clearance adjusted for glomerular filtration rate to accurately diagnose and manage nephrotic syndrome variants, particularly when proteinuria does not meet conventional thresholds but other clinical and pathological features are consistent with the syndrome.
